# Sesamol Inhibited Melanogenesis by Regulating Melanin-Related Signal Transduction in B16F10 Cells

**DOI:** 10.3390/ijms19041108

**Published:** 2018-04-07

**Authors:** Po-Yuan Wu, Ya-Jhen You, Yi-Jung Liu, Chien-Wei Hou, Chin-Sheng Wu, Kuo-Ching Wen, Chien-Yih Lin, Hsiu-Mei Chiang

**Affiliations:** 1 Department of Dermatology, China Medical University Hospital, Taichung 40402, Taiwan; wu.poyuan@gmail.com; 2 School of Medicine, China Medical University, Taichung 404, Taiwan; 3 Department of Cosmeceutics, China Medical University, Taichung 40402, Taiwan; jenn123gay@yahoo.com.tw (Y.-J.Y.); ella8175@hotmail.com (Y.-J.L.); cswu@mail.cmu.edu.tw (C.-S.W.); kcwen0520@mail.cmu.edu.tw (K.-C.W.); 4 Ph.D Program for Biotechnology Industry, China Medical University, Taichung 40402, Taiwan; 5 Department of Biotechnology and Pharmaceutical Technology, Yuanpei University of Medical Technology, Hsinchu 30015, Taiwan; rolis.hou@mail.ypu.edu.tw; 6 Department of Biotechnology, Asia University, Taichung 41354, Taiwan; yihlin@asia.edu.tw

**Keywords:** sesamol, melanogenesis, tyrosinase, microphthalmia-associated transcription factor (MITF), melanocortin 1 receptor (MC1R), glycogen synthase kinase 3 beta (GSK3β)

## Abstract

Melanin is synthesized through a series of interactions catalyzed by melanogenic enzymes such as tyrosinase, dopachrome tautomerase (tyrosinase-related protein-2; TRP-2), and tyrosinase-related protein-1 (TRP-1). Tyrosinase plays a key role in catalysing the initial and limiting steps of melanogenesis. The melanin that results from melanogenesis has the protective effect of absorbing ultraviolet radiation. However, overproduction of melanin, in addition to altering the appearance of skin, may lead to skin disorders such as melasma, solar lentigo, and postinflammatory hyperpigmentation. Previous studies have revealed that sesamol is a strong antioxidant and a free radical scavenger. In this study, we investigated the effects of sesamol on the regulation of melanogenesis and related mechanisms in B16F10 cells. The results indicated that sesamol inhibited tyrosinase activity and melanogenesis induced by α-melanocyte-stimulating hormone (α-MSH) in B16F10 melanoma cells. Sesamol decreased the protein level of melanocortin 1 receptor (MC1R), microphthalmia-associated transcription factor (MITF), tyrosinase, and TRP-1 by downregulating cyclic adenosine monophosphate (cAMP)/protein kinase A (PKA) pathways that had been activated by α-MSH. Sesamol increased glycogen synthase kinase 3 beta (GSK3β), protein kinase B (AKT), and extracellular signal-related kinase (ERK) phosphorylation, thus inhibiting the transcription of MITF. Sesamol also inhibited melanin synthesis and tyrosinase expression by modulating ERK, phosphoinositide 3-kinase (PI3K)/AKT, p38, and c-Jun amino-terminal kinase (JNK) signalling pathways. These results indicate that sesamol acted as a potent depigmenting agent.

## 1. Introduction

Skin colour is the manifestation of the progressive transfer of melanin to keratinocytes after melanogenesis in the melanosomes of melanocytes. Long-term ultraviolet (UV) exposure causes an abnormal increase in reactive oxygen species (ROS) generation, which induces melanogenesis to protect skin from the deleterious effects of UV irradiation and environmental pollutants [1,2,3]. However, excessive accumulation of melanin can influence appearance and cause pigmentation disorders such as freckles, age spots, solar lentigo, postinflammatory hyperpigmentation, melasma, and even melanoma [4,5].

Melanin synthesis comprises a series of complex processes regulated by various factors, including enzymes, proteins and hormones [6,7]. The limiting enzyme in melanogenesis is tyrosinase. l-tyrosine is transported from the extracellular space or intracellular generation through hydroxylation of l-phenylalanine, a precursor of tyrosine. Hydroxylation of tyrosine by tyrosinase produces l-3,4-dihydroxyphenylalanine (l-DOPA), which subsequently is oxidized to DOPAquinone [8]. DOPAquinone undergoes nonenzymatic intramolecular cyclization reaction to form leucochrome and then nonenzymatically oxidized to DOPAchrome. Tyrosinase-related protein-2 (TRP-2) will convert DOPAchrome to 5,6-dihydroxyindole-2-carboxylic acid (DHICA), which is catalysed by TRP-1 to form eulmelanin [9,10,11]. TRP-1 and TRP-2 stabilize and increase the activity of tyrosinase. Tyrosine and l-DOPA serve as substrates and intermediates of melanogenesis. In addition, they also act as inducers and positive regulators of the melanogenic pathway and of other cellular functions [12]. The cells surrounding melanocytes such as keratinocytes and fibroblasts may influence melanogenesis. α-Melanocyte-stimulating hormone (α-MSH) is a product of the processing of proopiomelanocortin (POMC), which, along with adrenocorticotropic hormone (ACTH), is produced in a regulated fashion by all resident skin cells, including keratinocytes, melanocytes, and fibroblasts, as well as by immune cells [7,13]. α-MSH binds to the melanocortin 1 receptor (MC1R) on melanocytes. Cyclic adenosine monophosphate (cAMP) stimulates the translocation of protein kinase A (PKA) into the nucleus, thus activating cAMP-response element-binding protein (CREB) [8,14]. Activation of cAMP increases microphthalmia-associated transcription factor (MITF) expression, which upregulates tyrosinase, TRP-1, and TRP-2 expression, ultimately promoting melanin synthesis in melanocytes [15].

Agents with antioxidant and tyrosinase inhibition activity can be used to prevent hyperpigmentation [15,16]. Sesame seeds contain strong antioxidants and are common food in Central and East Asia. Sesamol (3,4-(methylenedioxy)phenol), an active component in sesame seeds, is a potent antioxidant which scavenges free radicals [17,18]. Liu et al. have demonstrated that sesamol inhibits proliferation and promotes apoptosis of HepG2 cells [19]. Sesamol was also reported to prevent cardiovascular disease, coronary heart disease, and stroke [20]. The present study aimed to investigate the effect of sesamol on melanin synthesis in B16F10 cells. Sesamol’s effect on the regulation of cAMP/PKA, mitogen-activated protein kinase kinase (MEK)/extracellular signal-related kinase (ERK), protein kinase B (AKT)/glycogen synthase kinase 3 beta (GSK3β)/CREB, TRP-1, and MITF in melanin synthesis were also studied.

## 2. Results

### 2.1. B16F10 Cell Viability with Sesamol Treatment

**Figure 1:**
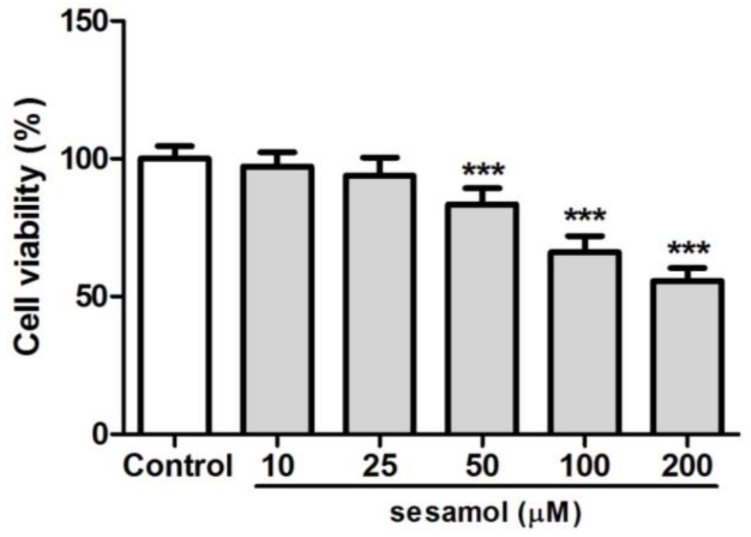
Cell viability (%) of B16F10 cells after 48 h of treatment with sesamol. The cell viability was below 80% at sesamol doses over 100 µM. Each value is presented as the mean ± SD. Significant difference with control group: *** *p* < 0.001.

After treatment with 10–200 µM sesamol for 48 h, cell viability was found to be higher than the 80% viability found at 10–50 µM. However, cell viability was 66.1% and 55.7% with 100 and 200 µM of sesamol treatment, respectively (Figure 1). Thus, the doses of sesamol used to study its effect on melanogenesis were 10–50 µM.

### 2.2. Sesamol Inhibited Melanin Biosynthesis in B16F10 Cells

**Figure 2:**
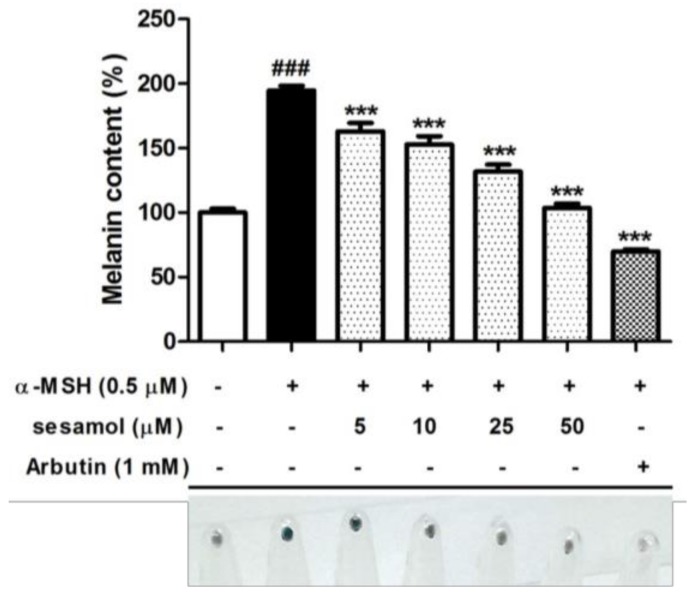
Melanin content (%) of B16F10 cells and cell pellets after 48 h of treatment with sesamol. Seasamol significantly inhibited melanin synthesis. Each value is presented as the mean ± SD. Significant difference versus control: ^###^ *p* < 0.001. Significant difference versus α-MSH-treated group: *** *p* < 0.001. Positive control: 1 mM arbutin.

Figure 2 shows the effects of sesamol on melanin content in B16F10 cells. The intracellular melanin content increased to 191.9% ± 3.5% after α-MSH treatment. Sesamol at doses higher than 5.0 µM significantly reduced the melanin content. After 50 µM sesamol treatment, melanin decreased to 90.1% ± 3.3% (Figure 2). The cell pellet was darker after α-MSH treatment, but it became lighter in the sesamol group. According to the results, sesamol significantly inhibited melanin biosynthesis.

### 2.3. Sesamol Inhibited Tyrosinase Activity in B16F10 Cells

**Figure 3:**
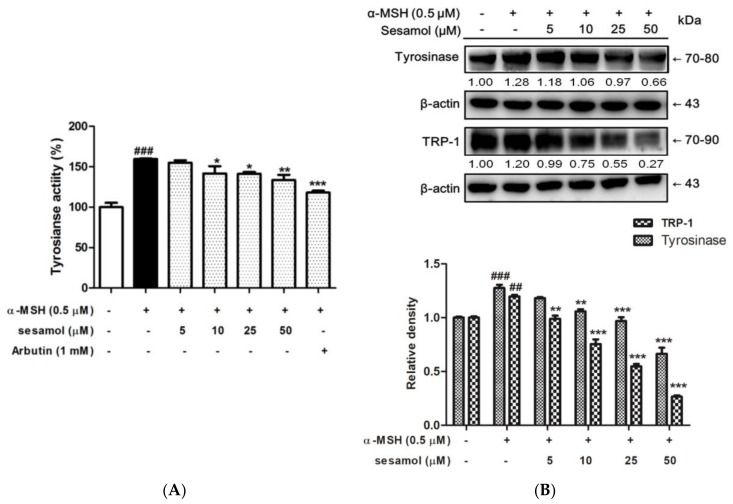
(**A**) Tyrosinase activity (%) of B16F10 cells after 48 h of treatment with sesamol. Sesamol inhibited tyrosinase activity in B16F10 cells. Each value is presented as the mean ± SD. Significant difference versus control: ^###^ *p* < 0.001. Significant difference versus α-MSH-treated group: * *p* < 0.05, ** *p* < 0.01, *** *p* < 0.001. Positive control: 1 mM arbutin. (**B**) Effect of sesamol on α-MSH-induced protein expression of tyrosinase and TRP-1 in B16F10 cells. Sesamol suppressed tyrosinase and TRP-1 protein levels. Each value is presented as the mean ± SD. Significant difference versus control: ^###^ *p* < 0.001, ^##^ *p* < 0.01. Significant difference versus α-MSH-treated group:** *p* < 0.01, *** *p* < 0.001.

Tyrosinase is the rate-limiting enzyme in melanin synthesis. Inhibition of tyrosinase activity is an efficient strategy in developing of antimelanogenic agents. Sesamol significantly inhibited tyrosinase activity in B16F10 cells (Figure 3A). The levels of tyrosinase activity were 159.6% ± 1.0% after α-MSH treatment, and became 154.9% ± 3.2%, 146.7% ± 5.7%, 141.3% ± 2.3%, and 133.6% ± 6.4% after treatment with 5, 10, 25, and 50 µM sesamol, respectively. The results indicated that sesamol inhibited tyrosinase activity in B16F10 cells.

### 2.4. Sesamol Inhibited Tyrosinase and TRP-1 Protein Expression in B16F10 Cells

In a protein expression assay using Western blotting, tyrosinase expression increased to 1.28-fold in the α-MSH group, and the protein expression of tyrosinase decreased to 1.18-, 1.06-, 0.97-, and 0.66-fold of the control value after 5–50 µM sesamol treatment for 48 h. Sesamol suppression of tyrosinase expression was dose dependent (Figure 3B). The results show that sesamol inhibited melanogenesis in B16F10 cells by suppressing tyrosinase activity and protein expression.

To understand the mechanism underlying sesamol’s regulatory effect on melanogenesis, the protein expression of TRP-1 was determined in B16F10 cells after their treatment with α-MSH and 5–50 µM sesamol for 48 h. The results show that at doses higher than 5 µM, sesamol significantly reduced the TRP-1 level (Figure 3B).

### 2.5. Sesamol Downregulated MC1R and MITF Expression

**Figure 4:**
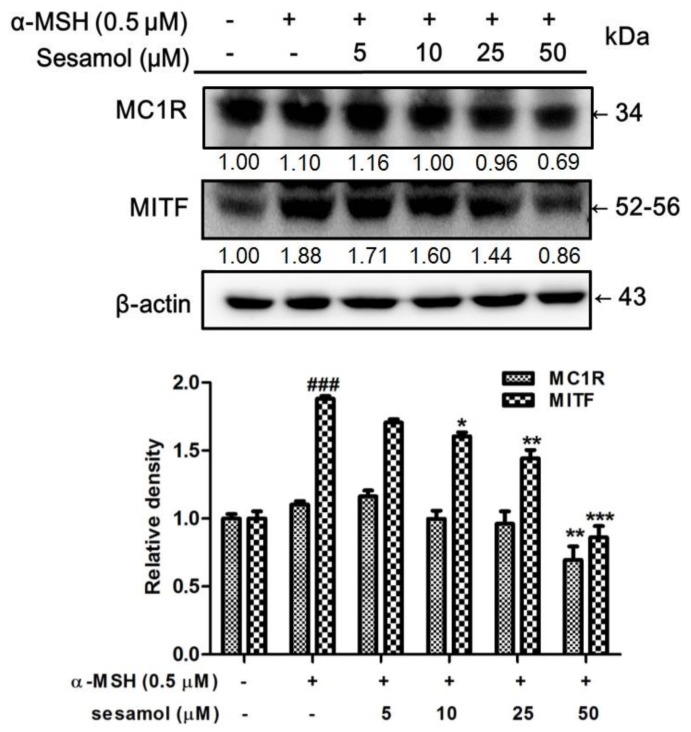
Effect of sesamol on α-MSH-induced protein expression of MC1R and MITF in B16F10 cells. Each value is presented as the mean ± SD. Significant difference versus control: ^###^ *p* < 0.001; Significant difference versus α-MSH-treated group: * *p* < 0.05, ** *p* < 0.01, *** *p* < 0.001.

MC1R expresses in melanocytes and is a key receptor in melanogenesis [21]. The expression of MC1R was found to be 1.10-fold for the control value. MC1R expression was 1.16-, 1.00-, 0.96-, and 0.69-fold with 5–50 µM sesamol treatments for 2 h (Figure 4). With 50 µM sesamol treatment, the protein expression of MC1R was significantly less than at the control value. MITF expression in B16F10 cells increased to 1.88-fold of the control in the α-MSH group (Figure 4). Inhibition of MITF expression varied with the sesamol treatment dose; at a concentration of 25 µM sesamol, MITF expression in the B16F10 cells was significantly downregulated (Figure 4).

### 2.6. Sesamol Inhibited Melanogenesis by Upregulating p-AKT and p-GSK3β Expression

**Figure 5:**
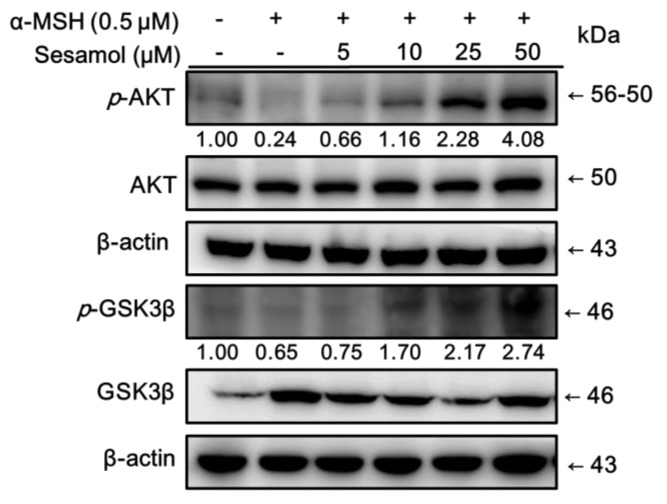

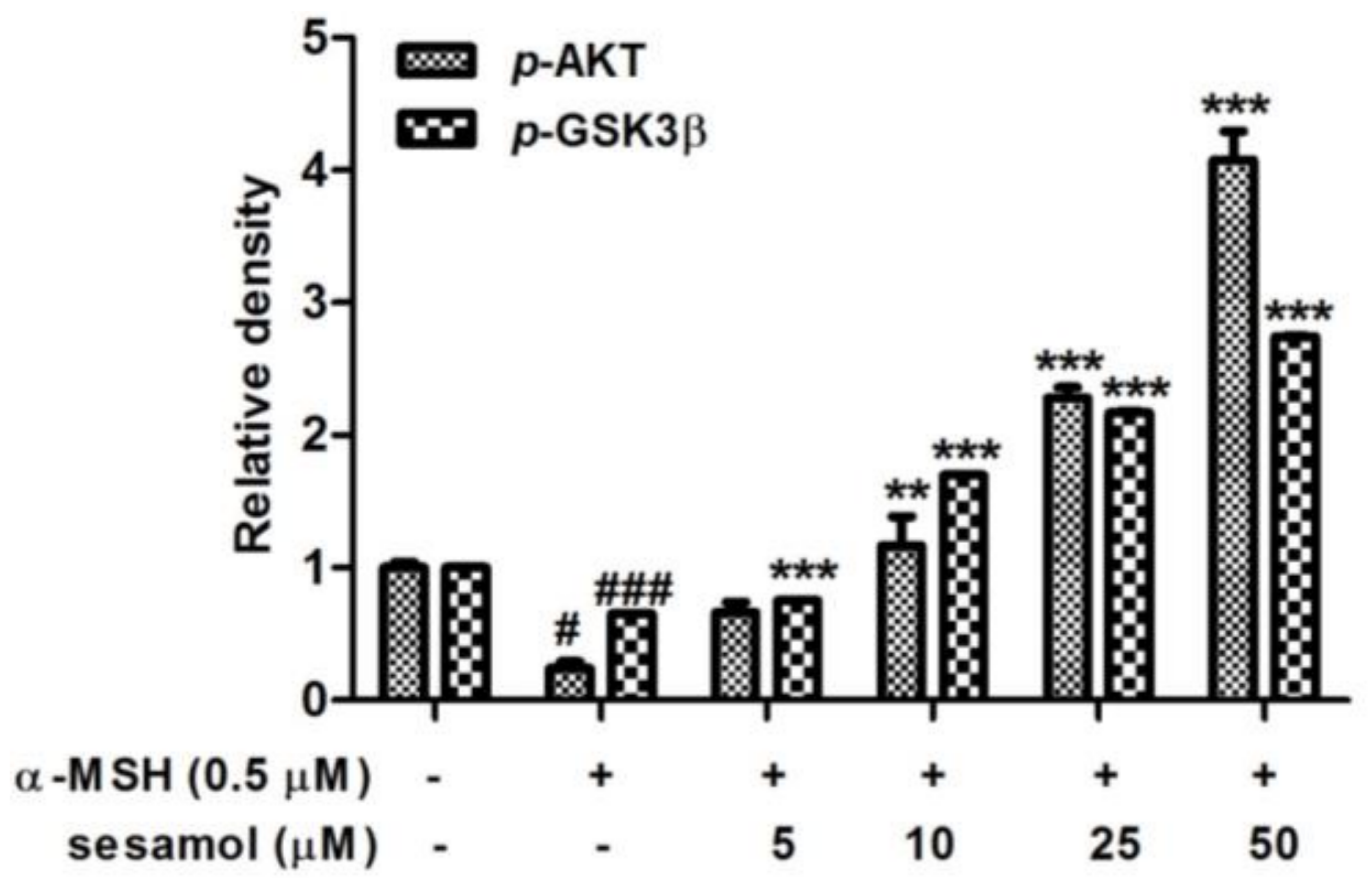
Effect of sesamol on α-MSH-induced protein expression of *p*-GSK3β and *p*-AKT in B16F10 cells. Each value is presented, in comparison with the control group, as the mean ± SD., ^#^ *p* < 0.05, and ^#^^##^ *p* < 0.001; and, in comparison with the α-MSH treated group, as ** *p* < 0.01, and ***p < 0.001.

Inhibition of the phosphorylation of AKT and GSK3β leads to the MITF activation resulting in melanin synthesis [22]. The expressions of AKT and GSK3β were determined to clarify the regulation of sesamol in this pathway. As shown in Figure 5, treatment with α-MSH significantly decreased *p*-AKT (0.24-fold of control) and *p*-GSK3β (0.65-fold of control) expression in B16F10 cells. Treatment with 10 µM sesamol for 48 h markedly increased *p*-AKT expression, and 48 h treatment with 5 µM of sesamol markedly increased *p*-GSK3β expression. These results indicate that sesamol activated the phosphorylation of AKT and GSK3β, leading to the downregulation of downstream signalling transduction, such as MITF expression, and resulting in inhibition of tyrosinase gene activity and expression, and, therefore, melanin synthesis.

### 2.7. Sesamol Upregulated p-ERK Expression

**Figure 6:**
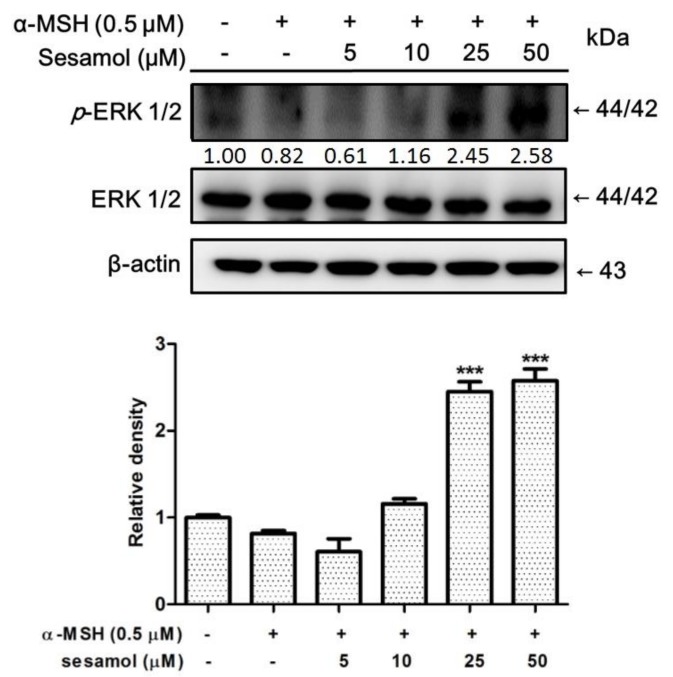
Effect of sesamol on α-MSH-induced protein expression of *p*-ERK in B16F10 cells. Each value is presented, in comparison with the α-MSH-treated group, as the mean ± SD. Significant difference versus α-MSH-treated group: *** *p* < 0.001.

Phosphorylation of ERK activates MITF to stimulus melanin synthesis. To understand the role of ERK in antimelanogenesis of sesamol, the expression of ERK was studied. After treatment with α-MSH, *p*-ERK expression in B16F10 cells became 0.82-fold compared with *p*-ERK expression in the control (Figure 6). Sesamol dose-dependently inhibited *p*-ERK expression in B16F10 cells after 48 h of treatment.

### 2.8. Effects of Sesamol on the Melanogenesis Signalling Pathway

To understand the mechanism underlying the depigmenting effect, we examined sesamol in cAMP/PKA, PI3K/AKT, MEK/ERK, p38, and JNK pathways along with H-89 (PKA/mitogen- and stress-activated protein kinase (MSK) inhibitor), LY 294002 (PI3K inhibitor), PD 98059 (ERK inhibitor), SB 203580 (p38 inhibitor), and JNK inhibitor II, respectively, for 48 h.

#### 2.8.1. Inhibition of Melanogenesis by Sesamol was Associated with PKA/MSK Regulation

**Figure 7:**
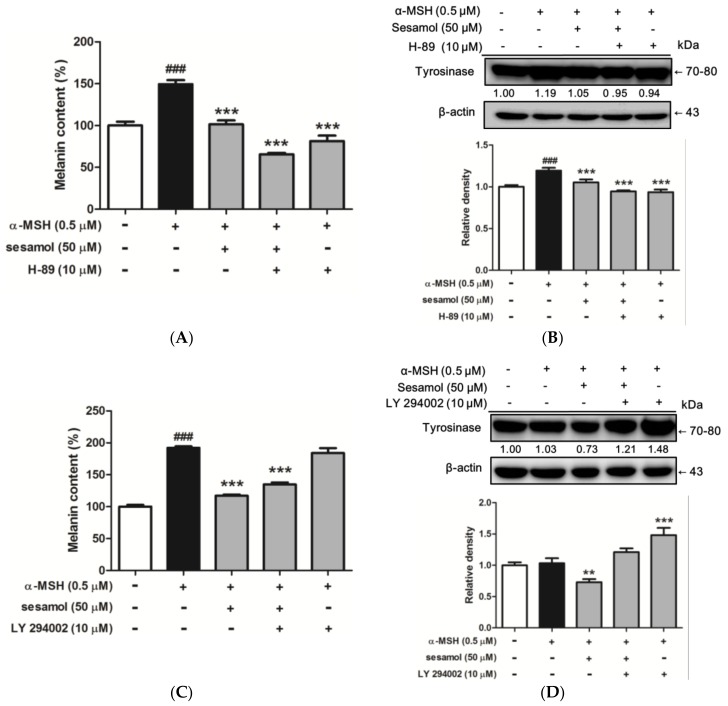
(**A**) Effect of sesamol and H-89 (PKA/MSK inhibitor) on melanin content (%) in α-MSH-treated B16F10 cells after 48 h. Each value is presented as the mean ± SD. ^###^ *p* < 0.001 (Significant difference versus control); *** *p* < 0.001 (Significant difference versus α-MSH treated group). (**B**) Effects of sesamol and H-89 (PKA/MSK inhibitor) on protein expression of tyrosinase in α-MSH-treated B16F10 cells after 48 h. Each value is presented as the mean ± SD. ^###^ *p* < 0.001 (Significant difference versus control); *** *p* < 0.001 (Significant difference versus α-MSH treated group). (**C**) Effects of sesamol and LY 294002 (PI3K inhibitor) on melanin content (%) in α-MSH-treated B16F10 cells after 48 h. Each value is presented as the mean ± SD. ^###^ *p* < 0.001 (Significant difference versus control); *** *p* < 0.001 (Significant difference versus α-MSH treated group). (**D**) Effects of sesamol and LY 294002 (PI3K inhibitor) on protein expression of tyrosinase in α-MSH-treated B16F10 cells after 48 h. Each value is presented as the mean ± SD. ** *p* < 0.01, *** *p* < 0.001 (Significant difference versus α-MSH treated group).

To determine whether inhibition of melanogenesis by sesamol was associated with the PKA/MSK pathway, B16F10 cells were incubated with 10 µM H-89 and 50 µM sesamol for 48 h. The melanin content and expression of tyrosinase was determined. As Figure 7A shows, the melanin content of B16F10 cells increased to 149.4% ± 4.9% relative to the control upon treatment with α-MSH. Subsequently, separate treatments with sesamol and H-89 reduced α-MSH-induced melanin content to 101.4% ± 4.5% and 81.5% ± 6.9% compared with that in the control, respectively (Figure 7A). In addition, cotreatment with sesamol and H-89 reduced melanin content to 65.7% ± 1.5% compared with that in the control.

As shown in Figure 7B, tyrosinase expression was 1.19-fold of the control after α-MSH treatment, but decreased to 1.05- and 0.94-fold, respectively, after sesamol and H-89 treatments. In addition, after cotreatment with sesamol and H-89, tyrosinase expression in B16F10 cells decreased to 0.95-fold of that in the control. These results indicate that PKA and MSK pathways may be involved in the antimelanogenic effect of sesamol.

#### 2.8.2. Sesamol Inhibited Melanogenesis by Inhibiting PI3K

To determine whether inhibition of melanogenesis by sesamol was regulated by the PI3K pathway, B16F10 cells were incubated with 10 µM LY 294002 and 50 µM sesamol for 48 h. The melanin content and expression of tyrosinase was determined. The melanin content of B16F10 cells increased to 192.3% ± 2.2% after α-MSH treatment, while separate incubation with sesamol and LY 294002 (PI3K inhibitor) reduced α-MSH-induced melanin content to 117.1% ± 1.9% and 183.9% ± 7.8%, respectively (Figure 7C). In addition, cotreatment with sesamol and LY 294002 reduced melanin content to 134.8% ± 3.1% compared with that in the control.

As shown in Figure 7D, tyrosinase expression was 1.03-fold of the control after α-MSH treatment, but 0.73- and 1.48-fold, respectively, after separate sesamol and LY 294002 treatments. In addition, cotreatment with sesamol and LY 294002 decreased tyrosinase expression to 1.21-fold of that in the control. The results indicate that the PI3K pathway may be involved in the antimelanogenic effect of sesamol.

#### 2.8.3. Sesamol Inhibited Melanogenesis by Inhibiting ERK

**Figure 8:**
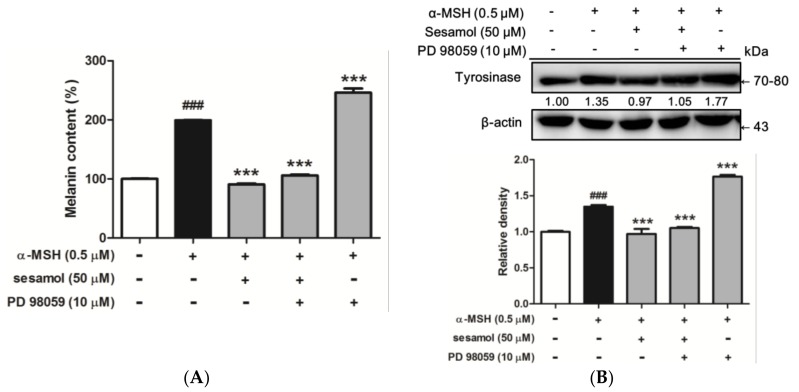
(**A**) Effects of sesamol and PD 98059 (ERK inhibitor) on melanin content (%) in α-MSH-treated B16F10 cells after 48 h. Each value is presented as the mean ± SD. ^###^ *p* < 0.001 (Significant difference versus control); *** *p* < 0.001 (Significant difference versus α-MSH treated group). (**B**) Effects of sesamol and PD 98059 (ERK inhibitor) on protein expression of tyrosinase in α-MSH-treated B16F10 cells after 48 h. Each value is presented as the mean ± SD. ^###^ *p* < 0.001 (Significant difference versus control); *** *p* < 0.001 (Significant difference versus α-MSH treated group).

To determine whether inhibition of melanogenesis by sesamol was regulated by ERK, B16F10 cells were incubated with 10 µM PD 98059 and 50 µM sesamol for 48 h. The melanin content and expression of tyrosinase was determined. The melanin content of B16F10 cells increased to 199.2% ± 0.6% after α-MSH treatment. Separate incubation with sesamol and 10 µM PD 98059 (ERK inhibitor) subsequently altered α-MSH-induced melanin content to 90.6% ± 1.6% and 246.2% ± 7.0%, respectively, compared with that in the control (Figure 8A). In addition, after cotreatment with sesamol and PD 98059, melanin content increased to 105.7% ± 1.4% compared with that in the control.

As shown in Figure 8B, the tyrosinase expression was 1.35-fold of that in the control after α-MSH treatment, but after separate sesamol and PD 98059 treatments, tyrosinase expression was 0.97- and 1.77-fold of the control, respectively. In addition, cotreatment with sesamol and PD 98059 increased tyrosinase expression to 1.05-fold of the control. The results indicate that the ERK pathway may be involved in the antimelanogenic effect of sesamol.

#### 2.8.4. Sesamol Inhibited Melanogenesis by Inhibiting p38

**Figure 9:**
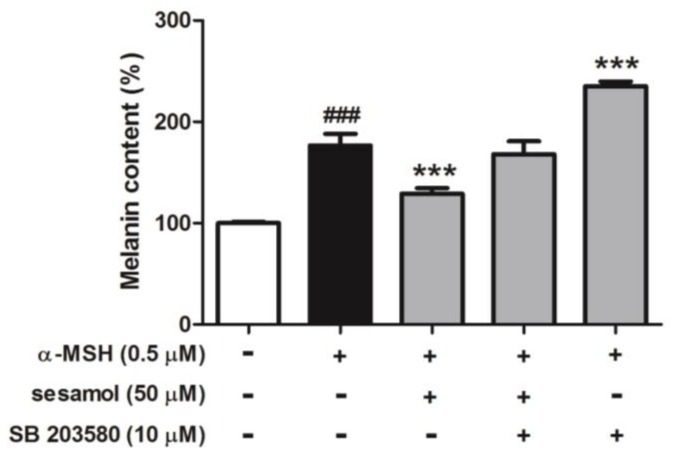
Effects of sesamol and SB 203580 (p38 inhibitor) on melanin content (%) in α-MSH-treated B16F10 cells after 48 h. Each value is presented as the mean ± S.D. ^###^ *p* < 0.001 (Significant difference versus control); *** *p* < 0.001 (Significant difference versus α-MSH treated group).

To determine whether inhibition of melanogenesis by sesamol was regulated by p38, B16F10 cells were incubated with 10 µM SB 203580 and 50 µM sesamol for 48 h. The melanin content of B16F10 cells increased to 176.6% ± 11.4% after α-MSH treatment, while separate incubation with sesamol and 10 µM SB 203580 (p38 inhibitor) regulated α-MSH-induced melanin content to 129.2% ± 5.4% and 235.0% ± 4.8%, respectively, compared with that in the control (Figure 9). In addition, after cotreatment with sesamol and SB 203580, melanin content was 168.2% ± 12.6% compared with that in the control.

#### 2.8.5. Sesamol Inhibited Melanogenesis by Inhibiting JNK

**Figure 10:**
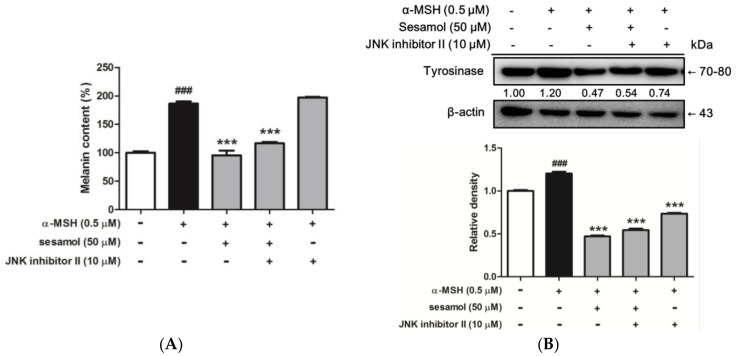
(**A**) Effect of sesamol and JNK inhibitor II on melanin content (%) in α-MSH-treated B16F10 cells after 48 h. Each value is presented as the mean ± SD. ^###^ *p* < 0.001 (Significant difference versus control); *** *p* < 0.001 (Significant difference versus α-MSH treated group). (**B**) Effects of sesamol and JNK inhibitor II on protein expression of tyrosinase in α-MSH-treated B16F10 cells after 48 h. Each value is presented as the mean ± SD. ^##^^#^ *p* < 0.001 (Significant difference versus control); *** *p* < 0.001 (Significant difference versus α-MSH treated group).

To determine whether inhibition of melanogenesis by sesamol was regulated by JNK, B16F10 cells were incubated with 10 µM JNK inhibitor II and 50 µM sesamol for 48 h. The melanin content and expression of tyrosinase was determined. The melanin content of B16F10 cells increased to 186.6% ± 3.6% after α-MSH treatment, while incubation with sesamol and 10 µM JNK inhibitor II separately regulated α-MSH-induced melanin content to 95.5% ± 8.0% and 197.1% ± 1.2% compared with that in the control, respectively (Figure 10A). In addition, after cotreatment with sesamol and JNK inhibitor II, melanin content was 116.7% ± 2.4% compared with that in the control.

Figure 10B shows that tyrosinase expression was 1.20-fold of the control value after α-MSH treatment, but the protein expression was 0.47- and 0.74-fold after separate sesamol and JNK inhibitor II treatment. In addition, cotreatment with sesamol and JNK inhibitor II increased tyrosinase expression to 0.54-fold of the control. The results indicate that the JNK pathway may be involved in the antimelanogenic effect of sesamol.

## 3. Discussion

Melanin synthesis is a series of oxidative reactions, and multiple enzymes are involved in these processes such as tyrosinase and TRP-1 and TRP-2. Tyrosinase, a copper-containing metalloenzyme, acts as the rate-limiting enzyme in the melanogenic pathway and plays a crucial role in initiating melanin synthesis [23,24]. Tyrosine is oxidized by tyrosinase to l-DOPA and DOPAquinone. DOPAquinone serves as the material to synthesize eumelanin or pheomelanin. Agents with antioxidative and tyrosinase-inhibiting properties have been previously recognized as hypopigmentation agents. Sesamol, because of its hydroxyl group, is a strong antioxidant and can inhibit biphenolase and monophenolase activity, such as that of tyrosinase, thereby inhibiting melanin synthesis in B16F10 cells [25]. A study reported that sesamol is a noncompetitive inhibitor of tyrosinase [25]. In our study, sesamol inhibited activity in both TRP-1 and tyrosinase, resulting in reduced melanin synthesis. TRP-1 is involved in tyrosinase stabilization and is critical to eumelanin production during the melanin synthesis process. Therefore, inhibition of TRP-1 may reduce the stability of tyrosinase [26]. In this study, sesamol inhibited α-MSH-induced melanogenesis in a dose-dependent manner. In addition, sesamol also inhibited tyrosinase activity and protein expression in B16F10 cells. These results are consistent with those of a previous study that demonstrated that at 25 and 50 µM, sesamol inhibited tyrosinase, TRP-1, and TRP-2 expression in melan-a cells and in melanin synthesis in zebrafish [27]. The reduction of tyrosinase, TRP-1, and TRP-2 protein levels may cause by inhibition of MITF, which is an important regulator of melanogenesis [28]. The results in this study indicate that sesamol inhibited α-MSH-induced MITF expression, leading to inhibition of melanin biosynthesis.

To further investigate the mechanism by which sesamol inhibits melanogenesis, we examined melanin-related protein regulation in PKA/cAMP/MITF/tyrosinase, ERK/tyrosinase, and PI3K/AKT/GSK3β/tyrosinase pathways. Melanin synthesis results from the following chain of events: keratinocytes secrete α-MSH which binds to MC1R, thereby activating adenylate cyclase to synthesise cAMP, which subsequently induces phosphorylation of CREB and activation of the MITF promoter [29]. Baek and Lee investigated that sesamol inhibited cAMP level in melan-a cells at 12.5 µM [27]. Our results indicate that sesamol inhibited α-MSH-induced CREB and MITF expression, ultimately attenuating melanin synthesis.

To understand the regulation of signal transduction underlying the depigmenting effect, the protein inhibitors were used to examine sesamol in PKA, PI3K and MAPK pathways. In our study, melanogenesis and tyrosinase activity were inhibited in B16F10 cells after the cells were cotreated with sesamol and H-89. One study reported that inhibited MSK1 activation may disrupt the synthesis of melanin [30]. MAPK pathway modulates the transcription activity of MITF and plays important role in melanin synthesis [31,32]. Extracts of *Astragalus membranaceus* increased the level of ERK phosphorylation and inhibited the production of melanin in a previous study [31]. Here, reduced expression of MITF was related to inhibiting phosphorylation of ERK. It was reported that sesamol induces phosphorylation of p38 and JNK, but not ERK in melan-a cells [27]. However, our study demonstrated that sesamol inhibited α-MSH-induced phosphorylation of ERK and, after PD 98059 cotreatment, reduced melanin content and tyrosinase activity. In addition, the results of this study indicate that sesamol’s regulation of p-38 and JNK signal transduction in B16F10 cells resulted in inhibition of melanin biosynthesis. Besides MAPK pathway, PI3K/AKT and GSK3β expression and activity cause MITF to bind to the target sequence and induce melanogenesis. Enhancing the activity of cAMP may inhibit PI3K/AKT and GSK3β expression and activity [33]. In our study, sesamol elevated the expression of *p*-AKT and *p*-GSK3β, possibly reducing MITF transcription to suppress tyrosinase gene expression and thus inhibiting melanin production and tyrosinase activity. Meanwhile, we found that through AKT and GSK3β activation and subsequent downregulation of MITF, CREB, tyrosinase, and TRP-1 production, sesamol inhibited α-MSH-induced hyperpigmentation in B16F10 cells.

## 4. Material and Methods

### 4.1. Chemicals and Materials

Sesamol (purity 98%), arbutin, l-DOPA, dl-dithiothreitol, H-89 dihydrochloride hydrate, LY294002, PD98059, and SB203580 were obtained from Sigma Chemical Co. (St. Louis, MO, USA). α-MSH was acquired from Merck (Darmstadt, Germany). An antibody recognizing MC1R was obtained from Millipore Corporation (Billerica, MA, USA). Antibodies recognizing AKT and phospho-AKT were obtained from GeneTex, Inc. (Irvine, CA, USA). Other primary and secondary antibodies were obtained from Santa Cruz Biotechnology (Santa Cruz, CA, USA). All other chemicals and reagents used in this work were high-quality and commercially obtainable.

### 4.2. Cell Cultures and Cell Viability Assay

B16F10 melanoma cells were cultivated in Dulbecco’s modified Eagle’s medium (GIBCO, Invitrogen Corporation, Grand Island, NY, USA) supplemented with 10% fetal bovine serum at 37 °C in an incubator with 5% CO_2_. Cells were harvested through trypsinisation. Cell viability was measured by using the 3-(4,5-dimethylthiazol-2-yl)-2,5-diphenyltetrazolium bromide (MTT) assay, as previously described [33].

### 4.3. Melanin Content and Tyrosinase Activity Assay in B16F10 Cells

The melanin content and tyrosinase activity of B16F10 cells were measured according to a method described in previous studies [34] and using an enzyme-linked immunosorbent assay (ELISA) reader (Tecan, Grodig, Austria) at 405 nm. Brifely, the B16F10 cells were cultured in six-well culture plates and incubated. The cells were treated with a medium containing α-MSH and various concentrations of sesamol for 48 h. NaOH (2N) was added to each well to lyse the cells, and the cells were then centrifuged. The amounts of melanin in the supernatant were spectrophotometrically measured at 405 nm

In the tyrosinase activity assay, the B16F10 melanoma cells were plated in a 24-well plate and treated with a medium containing α-MSH and various concentrations of sesamol for 48 h. The medium was removed and then 1% Triton X-100 mixed in 100 mM phosphate buffered saline was added. The mixture was frozen at −80 °C and thawed at room temperature, and then centrifuged. A freshly prepared substrate (15 mM l-DOPA) was then added to the supernatant and incubated. The absorbance of each well was subsequently read.

### 4.4. Western Blotting

The expression of melanogenesis-related proteins in B16F10 cells was detected through Western blotting, as previously described [33,35]. Cells were cultured in a 10-cm dish overnight. Subsequently, α-MSH and various concentrations of sesamol were added and incubated for 48 h or the indicated time. The protein content of cell lysates was assessed using the Bradford method. An equal amount of protein (20 µg) was loaded and separated on sodium dodecyl sulfate polyacrylamide gel electrophoresis (SDS-PAGE) gel. Blots on polyvinylidene difluoride (PVDF) membranes were blocked overnight with 5% (*w*/*v*) skimmed milk solution and with the following specific antibodies: anti-actin, anti-AKT, anti-*p*-AKT, anti-CREB, anti-*p*-CREB, anti-GSK3β, anti-*p*-GSK3β, anti-MITF, anti-TRP-1, and anti-tyrosinase. The membranes were washed with TBST and then incubated with G horseradish peroxidase. Bands were visualized using an Enhanced Chemiluminescence Plus Kit (Fujifilm, LAS-4000, Tokyo, Japan), and the density of the bands was determined using a densitometric program (MultiGauge v2.2, Fuji Photo Film Co., Tokyo, Japan).

### 4.5. Statistical Analyses

Values are presented as the mean ± standard deviation. The results presented in this paper are representative of at least three individual experiments. Differences in the effects of various treatments were compared using the Student’s *t*-test or ANOVA and, subsequently, Scheffe’s test. *p*-values of <0.05 were defined as significant.

## 5. Conclusion

This study demonstrated the antimelanogenic activity of sesamol in B16F10 cells. Sesamol exhibited antimelanogenic activity through the regulation of MEK/ERK, AKT/GSK3β, and CREB/MITF and resultant inhibition of tyrosinase and TRP-1 expression. Future applications of these findings may include use of sesamol in skin-whitening products. However, B16F10 is a rodent melanoma cell, and the results of this study have to be confirmed on human melanocytes in the future.

## Abbreviations

α-MSH	α-melanocyte-stimulating hormone
cAMP	cyclic adenosine monophosphate
CAPE	caffeic acid phenethyl ester
CREB	cAMP response element binding protein
p-CREB	phospho-cAMP response element binding protein
DHICA	5,6-dihydroxyindole-2-carboxylic acid
l-DOPA	l-dihydroxyphenylalanine
DMSO	dimethyl sulfoxide
FBS	fetal bovine serum
GSK3β	glycogen synthase kinase 3 beta
MC1R	melanocortin 1 receptor
MITF	microphthalmia-associated transcription factor
MTT	3-(4,5-dimethylthiazol-2-yl)-2,5-diphenyltetrazolium bromide
PKA	protein kinase A
TRP-1	tyrosinase-related protein-1
TRP-2	tyrosinase-related protein-2
